# Genetic Adaptation of *Achromobacter* sp. during Persistence in the Lungs of Cystic Fibrosis Patients

**DOI:** 10.1371/journal.pone.0136790

**Published:** 2015-08-27

**Authors:** Winnie Ridderberg, Signe Maria Nielsen, Niels Nørskov-Lauritsen

**Affiliations:** 1 Department of Clinical Microbiology, Aarhus University Hospital, Aarhus, Denmark; 2 Institute of Clinical Medicine, Health, Aarhus University, Aarhus, Denmark; Ghent University, BELGIUM

## Abstract

*Achromobacter* species are increasingly isolated from the respiratory tract of cystic fibrosis patients and often a chronic infection is established. How *Achromobacter* sp. adapts to the human host remains uncharacterised. By comparing longitudinally collected isolates of *Achromobacter* sp. isolated from five CF patients, we have investigated the within-host evolution of clonal lineages. The majority of identified mutations were isolate-specific suggesting co-evolution of several subpopulations from the original infecting isolate. The largest proportion of mutated genes were involved in the general metabolism of the bacterium, but genes involved in virulence and antimicrobial resistance were also affected. A number of virulence genes required for initiation of acute infection were selected against, e.g. genes of the type I and type III secretion systems and genes related to pilus and flagellum formation or function. Six antimicrobial resistance genes or their regulatory genes were mutated, including large deletions affecting the repressor genes of an RND-family efflux pump and a beta-lactamase. Convergent evolution was observed for five genes that were all implicated in bacterial virulence. Characterisation of genes involved in adaptation of *Achromobacter* to the human host is required for understanding the pathogen-host interaction and facilitate design of future therapeutic interventions.

## Introduction

*Achromobacter* species are environmental bacteria innately resistant to many antibiotics [1]. *Achromobacter* sp. are increasingly isolated from patients with cystic fibrosis (CF)[2–4] and are recognised as important emerging pathogens in CF. Longitudinal studies have shown that clonally related isolates are repeatedly recovered from respiratory secretions of CF patients, indicating persistence of a single linage during chronic infection [4–7]. During establishment and maintenance of chronic infection, bacteria are subjected to numerous selective pressures arising from host immune system, co-infecting microorganisms and antimicrobial treatments [8, 9]. Adaptive evolution of CF pathogens *Pseudomonas aeruginosa* and *Staphylococcus aureus* during chronic infection include altered virulence, formation of biofilms, switch to small-colony variants and occurrence of hypermutable isolates [8, 10–13]. Short-term adjustments are believed to be the result of alterations in gene expression, whereas long-term adaptation is the result of loss-of-function mutations, deletions, insertions, inversions and recombination. Beneficial mutations are fixed by natural selection, giving rise to clonal diversification within the host [8, 11, 14].

In this study we performed a comparative genome analysis of clonal lineages of *Achromobacter* sp. from five patients with CF, in order to investigate the genetic adaptation of *Achromobacter* to the human host. The study was based on genome sequences of 15 longitudinally collected isolates originating from five CF patients chronically infected with *Achromobacter* sp.

## Materials and Methods

### Isolates

Serial isolates of *Achromobacter* sp. were obtained from airway secretions from five CF patients at the CF Centre at Aarhus University Hospital, Denmark. The five patients had been affiliated with the CF centre in Aarhus for up to 15 years, and all previous sputum samples had been culture-negative for *Achromobacter* sp. At the CF centre, airway secretions from patients are routinely cultured at monthly intervals. Incipient isolates (first-time detection) of *Achromobacter* sp. and two consecutive isolates (1–3 years apart) from each patient were analysed. Isolates were cultured on 5% blood agar at 35°C. Identification to genus was performed with matrix-assisted laser desorption/ionization time-of-flight (MALDI Biotyper, Bruker, Bremen, DE) and confirmed by 16S rRNA gene sequencing. Species identification of isolates was performed with Multilocus Sequence Analysis (MLSA) [15, 16]. The clonal relationship of serial isolates was confirmed with Pulsed-Field Gel Electrophoresis (PFGE) as described by Turabelidze et al. [17] using restriction enzyme *XbaI*. Electrophoresis was carried out for 22 hours at 6 V/cm with pulse-times ranging from 5 s to 35 s using the CHEF-DR II system (Bio-Rad). Restriction patterns were interpreted according to the criteria of Tenover et al. [18]. Each of the five patients was infected with a unique strain not detected in any other patient from the centre [4]. The gel picture is shown in supporting information S1 Fig

### Genome Sequencing and Analysis

Genomic DNA was extracted using DNeasy Blood and Tissue Kit, Qiagen, following instructions for Gram-negative bacteria, but washing twice with buffer AW2. Genomic libraries facilitating indexed paired-end sequencing were prepared using a TruSeq DNA Sample Prep kit (Illumina, Part #15026486 Rev. C, July 2012, gelfree protocol).

Whole-genome sequencing was performed on the Illumina HiSeq 2000 platform, generating 101-bp paired-end reads. An average sequence coverage depth of 372–475X was obtained. Reads from incipient isolates from each of the five patients were *de novo* assembled using CLC Genomics Workbench 7.5 (www.clcbio.com) using default settings with adapter-trimming and quality filter of 0.05 (CF2-5) or 0.01 (CF1). *De novo* assembled genomes were annotated using Rapid Annotations using Subsystems Technology (RAST)[19, 20]. Each *de novo* assembly was used as the reference genome sequence to map reads from consecutive isolates using the BWA-mem algorithm [21]. Sequence reads were trimmed using Trimmomatic [22] prior to mapping, removing adapter-sequences and bases of average phred quality less than 20, using a sliding window of four. Single Nucleotide Polymorphisms (SNP) and small indels were called using Platypus with default settings [23]. Only high quality SNPs supported by a minimum of 10 reads were retained. Large structural variants were called using Pindel [24]. Filtering and annotation of variants was performed with SnpSift and SnpEff, respectively [25, 26]. Provean was used to predict the functional effect of non-synonymous SNPs [27]. All variants were visually inspected in Artemis [28].

### Phenotypic characterisation

Antimicrobial susceptibility of isolates was determined by broth microdilution using Sensititre ESBL Plates (TREK Diagnostic Systems, Cleveland, OH).

Biofilm formation (chrystal violet microtitre PEG-lid assay) was assessed in 96 well polystyrene microtitre plates with PEG-Lids (Nunc-Immuno TSP). The protocol was modified from O’Toole et al. [29] and Harrison et al. [30]. Briefly, isolates were grown in liquid culture (Brain Heart Infusion Broth, Fluka) overnight and adjusted to OD_600nm_ = 0.1, corresponding to approximately 10^6^ CFU/ml. A 160 μL aliquote was transferred to micro titre wells, PEG-lids were inserted and incubated for 24 h at 37°C. Each strain was tested in eight wells per plate, and plates were tested in triplicate. After incubation PEG-lids were washed in water and left to dry for 30 min. Attached cells and extracellular material were stained with 1% crystal violet for 15 min. PEG-lids were washed three times in water and left to dry. Finally, PEG-lids were submerged in 96% ethanol for 15 min to extract the crystal violet. Biofilm formation was quantitated by measuring OD_585nm_ using a BioTek Power wave XS2 plate reader (Holm&Halby).

Amino acid auxotrophy was assessed by plating isolates onto Müeller Hinton Agar (Fluka) and Davis Minimal Agar (Fluka). Approximately 10,000 CFU were plated on agar plates and incubated at 37°C for one week. Strains unable to grow on Davis Minimal Agar were considered auxotrophic. Specific amino acid requirements were identified by plating strains onto Davis Minimal Agar supplemented with either single amino acids or combinations of 19 amino acids as specified in the text [31]. Agar plates were prepared using Davis Minimal Agar containing 20 μg/mL of specified amino acids (L-forms, Fluka). Control agars, Müller Hinton Agar and Davis Minimal Agar without amino acids, were included in all tests.

### Ethics Statement

Bacterial strains are routinely preserved for epidemiological purposes in agreement with local guidelines. The study was approved by the Danish Data Protection Agency.

## Results and Discussion

We sequenced the genomes of 15 *Achromobacter* sp. isolates—three isolates (a, b, c) from each of five patients with CF: three isolates of *A*. *ruhlandii*, three isolates of *A*. *xylosoxidans*, and nine isolates of *A*. *insuavis* (Table 1). We compared incipient isolates (a) with isolates collected during the following 2–4 years (b and c), to investigate adaptation to the CF lung and the evolutionary paths of different clonal lineages.

**Table 1:** Overview of patients and isolates.

Patient	Isolate	Isolation Year	Species
CF1	9557–07	2007	*A*. *ruhlandii*
	4616–09	2009	
	4951–12	2012	
CF2	19840–07	2007	*A*. *xylosoxidans*
	19373–08	2008	
	8663–11	2011	
CF3	10719–08	2008	*A*. *insuavis*
	13140–09	2009	
	13665–10	2010	
CF4	14174–08	2008	*A*. *insuavis*
	10317–09	2009	
	1036–12	2012	
CF5	15059–09	2009	*A*. *insuavis*
	23156–10	2010	
	5764–12	2012	

General features of draft genomes of incipient isolates are shown in Table 2. Draft genomes of *A*. *insuavis* varied between 6.7–6.8 Mb, while draft genomes of *A*. *xylosoxidans* and *A*. *ruhlandii* were slightly smaller (6.5 Mb). All genomes had a relatively high GC-content, 67.6–68.3%, which is comparable to that of other published *Achromobacter* sp. genomes [32, 33].

**Table 2:** General features of draft genomes of incipient isolates.

Patient	Isolate	Genome Size (bp)	GC-content (%)	No. Contigs	Contig N50	Coverage Depth (x)	No. Coding Sequences*
CF1	9557–07	6,572,583	67.6	99	188600	475	6031
CF2	19840–07	6,500,112	67.6	513	39898	373	5939
CF3	10719–08	6,720,201	68.3	453	47841	372	6038
CF4	14174–08	6,831,652	68.2	647	28393	420	6090
CF5	15059–09	6,730,986	68.3	497	36413	442	6049

*Number of coding sequences as determined by RAST.

Sequences from the two consecutive isolates from each patient were mapped against the draft genomes to an average coverage depth of 416X. Reads were evenly distributed along reference sequences. For all data we obtained high sequence coverage breadth (99.4–99.9%), enabling almost complete detection of mutational variants between serial isolates.

We detected a total of 460 mutations in the genomes of the ten subsequent isolates relative to the five incipient isolates, 378 in coding sequences and 82 in non-coding sequences. Mutational events are summarised in Table 3. The majority of mutations in coding sequences were SNPs, and 81% of these were non-synonymous. Approximately half of non-synonymous SNPs were predicted to affect protein function as evaluated by Provean. Thirteen deletions and four insertions affecting coding sequences were also detected. Only a subset of variants detected in isolates b were also found in isolates c, suggesting a heterogeneous bacterial population, where distinct sub-populations evolve from the original infecting isolate during the course of colonisation. The number of mutational events detected in each of the five series of isolates showed large variation, with five mutations in isolates from patient CF3 to 337 mutations in isolates from patient CF5.

**Table 3:** Number of detected mutations.

Patient	Genome^1^	No. SNPs	No. Synonymous SNPs	No. Non-synonymous SNPs	No. Deletions	No. Insertions	No. Intergenic Variants^2^	Total No. Variants
CF1	b	26	3	23	1	1	2	
	c	10	2	8	1	0	3	
	b+c	8	2	6	0	0	0	
	Total	44	7	37	2	1	5	52
CF2	b	5	0	5	0	0	3	
	c	17	3	14	2	0	2	
	b+c	9	2	7	1	0	3	
	Total	31	5	26	3	0	8	42
CF3	b	0	0	0	0	0	0	
	c	0	0	0	0	0	2	
	b+c	2	1	1	0	0	1	
	Total	2	1	1	0	0	3	5
CF4	b	2	1	1	0	0	0	
	c	10	0	10	1	1	1	
	b+c	4	1	3	1	0	4	
	Total	16	2	14	2	1	5	24
CF5	b	69	10	59	2	0	19	
	c	149	31	118	4	1	34	
	b+c	50	11	39	0	1	8	
	Total	268	52	216	6	2	61	337
		361	67	294	13	4	82	460

^1^b, Mutations only found in isolate b compared to isolate a; c, mutations only found in isolate c compared to isolate a; b+c, mutations found in both isolates b and c compared to isolate a.

^2^number of mutations in non-coding sequences.

Table 4 lists the number of mutated genes according to functional class for each of the five clones (a complete list of mutated genes can be found in S1 Table). The majority of affected genes were involved in the general metabolism, but genes involved in transcription and translation, virulence, cell wall and capsule and stress response were also frequently mutated.

**Table 4:** Mutated genes (N = 311) grouped according to functional category (RAST). The number of mutated genes in each category is listed for clones from each of the five patients.

Functional Category	CF1	CF2	CF3	CF4	CF5	Total
Metabolism	22	15	1	8	123	169
Transcription and translation	7	6	0	5	19	37
Virulence, disease and defence	7	1	0	1	21	30
Hypothetical protein	2	1	0	1	17	21
Cell wall and capsule	0	4	0	2	10	16
Iron acquisition and metabolism	0	0	0	0	15	15
Stress response	1	1	0	0	5	7
DNA repair	0	0	0	0	6	6
Antibiotic resistance	0	1	0	0	3	4
Nitrogen metabolism	1	0	0	0	2	3
Phages, prophages, transposable elements, plasmids	0	0	0	0	3	3
**Total**	**40**	**29**	**1**	**17**	**224**	**311**

### General metabolism

At least seventeen genes were directly involved in amino acid synthesis. We observed a 79 bp deletion of the 5’-end of the tryptophan-synthase-beta-chain-encoding gene, involved in the synthesis of tryptophan [34]. The gene encoding threonine dehydratase contained a non-synonymous substitution leading to an Ala86Glu amino acid substitution, which was predicted to be deleterious for protein function. Threonine dehydratase converts L-Threonine to ammonia and alpha-ketobutarate—a precursor of L-isoleucine [35]. The gene encoding cystathionine gamma-synthase contained a non-synonymous substitution resulting in a Tyr77Cys replacement, predicted to impact protein function. Cystathionine gamma-synthase is involved in the synthesis of methionine from cysteine [36]. Similarly, the genes whose products take part in the biosynthesis of glycine (glycine cleavage system [37]), serine (D-3-phosphoglycerate dehydrogenase [38]) and aromatic amino acids phenylalanine, tyrosine and tryptophan (3-dehydroquinate synthase [39]) contained non-synonymous SNPs. Amino acid auxotrophy is well described during chronic *P*. *aeruginosa* CF infections [40]. Bacteria employ down-regulation of metabolic pathways as a mean of saving energy. Amino acid concentrations are elevated in CF sputum, which ensure that bacteria have sufficient supply of selected amino acids in the surrounding environment [40] and make synthesis of these redundant.

All isolates from patients CF2 and CF4 exhibited amino acid prototrophy when tested on Davies mimimal agar. Isolates b and c from patients CF1 and CF5 were auxotrophs and were subjected to further analyses to identify their specific amino acid requirements. Isolate CF1-b contained the substitution Ala86Glu in the threonine dehydratase. We did not find isolate CF1-b to be dependent on threonine for growth. However, we did observe that this isolate required leucine, isoleucine and valine to support growth on minimal agar. This is consistent with our finding of a Leu103Pro substitution in the branched amino acid transport protein, LivF, which is predicted to be deleterious for protein function. Analysis on selective agar of isolates CF5-a, CF5-b and CF5-c showed no specific amino acid requirements for strain CF5-b. Strain CF5-c only grew on minimal agar supplemented with glycine, alanine, valine and leucine. Dependence on valine and leucine for growth could be caused by dysfunction of the branched amino acid binding protein as a result of the observed Gly254Ser substitution. The remaining identified amino acid substitutions in proteins related to amino acid metabolism in isolate CF5-c do not appear to influence the amino acid requirements for this isolate.

Other genes not essential for survival in the new environment were also eliminated, e.g. genes conferring arsenic resistance (arsenical resistance protein and glutathione-S-transferase) [41] and the endo-type 6-aminohexanoate oligomer hydrolase rendering bacteria able to degrade nylon oligomers [42].

### Cell wall and capsule

Genes affecting formation and maintenance of cell wall, capsule and o-antigen were mutated in many isolates. Genes *rmlA* (Glucose-1-phosphate thymidylyltransferase) and *rmlC* (dTDP-4-dehydrorhamnose 3,5-epimerase) involved in biosynthesis of precursors of L-rhamnose, a key component of cell wall, both contained nonsynonymous SNPs. The DNA-binding capsular synthesis response regulator, *rcsB*, and an O-antigen acetylase also carried presumably deleterious mutations. Common for these genes are their presumptive roles in biofilm formation, based on studies on *Escherichia coli* [43], *P*. *aeruginosa* [44], *Stenotrophomonas maltophilia* [45, 46] and *Salmonella typhimurium* [47].

Isolates from patients CF2, CF4 and CF5 contained mutations in genes involved in biofilm formation. Biofilm formation of these nine strains was quantitated by the chrystal violet assay. In general, subsequent isolates showed reduced biofilm formation compared to incipient isolates. Isolates CF2-b and CF2-c showed a reduction in biofilm production of 11% and 18%, respectively, compared to isolate CF2-a. Both isolates were found to contain amino acid substitutions in RmlA, which could account for the observed reduction in biofilm production. Isolates CF4-b and CF4-c showed a reduction in biofilm production of 79% and 65% respectively, compared to incipient isolate CF4-a, and isolates CF5-b and CF5-c showed a 54% and 75% reduction, respectively, in biofilm production when compared to incipient isolate CF5-a. Isolate CF4-b harboured a mutated O-antigen acetylase, which may explain the observed decrease in biofilm formation. However, the O-antigen acetylase was not found to be mutated in isolate CF4-c, the latest isolate from CF4, and cannot account for the diminished biofilm formation of this isolate. RlmC and the diguanylate cyclase harboured amino acid substitutions in isolate CF5-b, and LapE was mutated in isolate CF5-c. Impairment of these proteins could account for the observed decrease in biofilm production by the two isolates.

In *P*. *aeruginosa*, chronic infection is also associated with loss of motility due to lack of pili and flagellum. Mutations of *rpoN* were shown to account for lack of both pili and flagellum in most cases [48], and indeed we found *rpoN* to contain a non-synonymous SNP in a single isolate. In addition, several other genes related to formation of pili and flagellum also carried SNPs and the gene encoding the flagellar motor rotation protein (*motB*) was observed to contain a 13 bp insertion, leading to a frame shift and loss of stop codon.

### Antimicrobial resistance

Strains of *Achromobacter* sp. are resistant to a wide range of antibiotics [49]. Resistance has been shown to be mediated both trough naturally occurring and acquired beta-lactamases, and efflux pumps [50–52].

Several genes related to multidrug resistance were mutated during the course of infection in our CF patients. The gene encoding a membrane fusion protein of an RND-family multidrug efflux pump contained a nonsynonymous SNP leading to amino acid substitution Pro351Leu. In another patient, the gene encoding a transcriptional repressor (tetR family) of an RND-family multidrug efflux pump contained a 162 bp-deletion. This may lead to constitutive expression of the operon encoding the genes of the tripartite efflux-system. Mutations in three beta-lactamase-encoding genes or their regulators were disclosed. A LysR-family transcriptional regulator located immediately upstream to a beta-lactamase-encoding gene was shown to harbour a non-synonymous SNP, resulting in Asp274Ala substitution. In a class C beta-lactamase gene we found a point mutation leading to the substitution of Pro202Leu, and finally, we observed a 1225-bp-deletion of the Murein-DD-endopeptidase-encoding gene. Murein-DD-endopeptidase is involved in synthesis and recycling of peptidoglycan, and intermediates of these processes have been shown to induce beta-lactamase production in gram-negative bacteria [53]. Furthermore, 270 bp of the transcriptional repressor located immediately upstream of the murein-DD-endopeptidase and a class D beta-lactamase gene (*bla*_OXA-114_) was also deleted.

The functional implications of the observed mutational events in antimicrobial resistance-related genes are not easily predicted, as antimicrobial resistance is mediated by multiple mechanisms of which only a few have been described for *Achromobacter* sp. [50–52]. Furthermore, the natural substrates of antimicrobial resistance mechanisms may be host-encoded antimicrobial compounds or toxic compounds occurring in the natural environment of the pathogen. Alterations in genes implicated in antimicrobial resistance may therefore be a response towards antibiotic pressure or simply down regulation of a mechanism that is no longer needed [54].

Antimicrobial susceptibility testing of the 15 isolates showed only few alterations in minimum inhibitory concentration (MIC) values that could possibly be linked to the observed mutations. Isolate CF2-c, in which the transcriptional repressor of a beta-lactamase encoding gene was partially deleted, had an increased MIC for meropenem (4 g/mL) compared to preceding isolates CF2-a and CF2-b (1 g/mL). MIC for meropenem was also increased for isolate CF4-c (2 g/mL), compared to preceding isolates CF4-a and CF4-b (1 g/mL). However, both isolates CF4-b and CF4-c contained the Asp274Ala substitution in the transcriptional regulator upstream of a beta-lactamase encoding gene.

In all other cases we were unable to correlate observed amino acid substitutions with measured MIC values.

### Iron uptake systems

Iron is essential for numerous biochemical processes. The ability to take up and utilise iron in various forms is important for the ability of pathogenic bacteria to invade and survive in their host. Iron uptake systems are therefore tightly coupled to the virulence of bacteria, and most bacteria employ several different iron uptake systems [55–57]. We found two genes of the hemin uptake locus to be mutated. The heme uptake transmembrane sensor gene, essential for expression of all genes in this locus [55], contained a 15 bp deletion in one isolate, and in another isolate the gene encoding the periplasmic hemin-binding protein contained a non-synonymous point mutation. Three genes related to siderophore-dependent iron uptake were shown to contain non-synonymous SNPs, in addition to a point mutation in a gene encoding a ferric iron ABC transporter. Several studies have shown reduced virulence of bacteria due to mutations in the iron uptake systems [58, 59].

### Other virulence-related traits

A number of other genes implicated in bacterial virulence were also found to contain non-synonymous SNP. The gamma glutamyltranspeptidase gene [60], the nitrous reductase gene *nosR* [33, 61], and the copper resistance gene *copG* [62] are necessary for defence against host immune system at initial infection. In addition, genes of the type I and type III secretion systems, *lapE* [63], *yopD* [64] and a patatin-encoding gene [65] also harboured mutations.

### Convergent evolution

Only five genes were mutated in more than one isolate. However, these genes have all been proposed to be associated with virulence or adaptation to stressful environments, suggesting that they were subject to positive selection as beneficial mutations. Gamma glutamyltranspeptidase is a virulence factor [60], while *relA* (GTP pyrophosphokinase) [66, 67] and *rsbW* (serine-protein kinase) [68, 69] are regulators of virulence factors. *kdpD* encodes an osmosensitive K+ channel histidine kinase involved in the high-affinity K+ uptake system used under extreme K+ limited conditions. K+ is an essential metabolic regulator important for bacterial adaptation to stressful environments [70]. Several mutations of *kdpD* have been shown to confer constitutive expression of the kdp-operon encoding the high-affinity K+ transport system [71]. The fifth gene to show convergent evolution encodes the cytochrome *o* ubiquinol oxidase subunit I. Cytochrome *o* oxidase is the main terminal oxidase of the electron transport chain under highly aerobic conditions. When oxygen supply is limited, an alternative terminal oxidase, cytochrome *d* oxidase, is synthesised [72]. Oxygen levels may become limited during the course of chronic infection and due to biofilm formation, making cytochrome *o* oxidase unnecessary for bacterial growth [73].

The small number of genes showing convergent evolution might be due to the limited sample size. However, divergent evolution is supported by the demonstration of several subpopulations co-evolving from the original infecting isolate. Apparently, the adaptation of *Achromobacter* sp. to the CF lung can involve a multitude of genes and do not require particular mutations. To what extent the strain-specific adaption is determined by the infecting clone, antimicrobial treatments, co-infecting microorganisms, or host factors, is not clear at present.

### Elevated accumulation of mutations

The number of accumulated mutations in isolates from patient CF5 was strikingly larger than observed among isolates of any of the remaining four patients. Hypermutable isolates of *P*. *aeruginosa* [74], *S*. *aureus* [75] and *H*. *influenzae* [76] are frequently isolated from CF patients. The mutator phenotypes of these species are attributed to inactivation of the methyl-directed mismatch repair (MMR) system, caused by mutation. Hypermutation is believed to contribute to the bacterial adaptation to new and changing environments. The excess of mutations in isolates from CF5 was predominantly (95%) due to transitions and not transversions, which is characteristic of hypermutation caused by mutation of *mutS* or *mutL* of the MMR-system [77]. We did not find mutations in the MMR-system in isolates of patient CF5, but we discovered a 112-bp-deletion of a gene encoding a DNA alkylation repair enzyme. However, the deletion was encountered only in isolate c, and thus cannot fully explain the elevated accumulation of mutations. Five genes implicated in DNA repair contained nonsynonymous SNPs (one in isolate b and four in isolate c). However, the resulting amino acid changes were all predicted to be neutral. The incipient isolate (a) may have carried mutations in the MMR-system and phenotypically be hypermutable. Comparison of mutS and mutL protein sequences from the three *A*. *insuavis* incipient isolates revealed two amino acid differences between mutS from CF5 and the two other isolates (CF3 and CF4), and one amino acid difference between mutL from CF5 and the other two isolates (CF3 and CF4). Provean predicted all three amino acid changes to neutral. Further studies are required to clarify if hypermutation drives accumulation of mutations in isolates from patient CF5.

## Conclusions

Adaptation of *Achromobacter* sp. to the CF lung involves inactivation of redundant metabolic functions, loss of virulence factors, and putative alterations in the expression of antimicrobial resistance genes. Virulence factors, required for initiation of acute infection, are selected against during chronic infection. The observation that the large majority of mutations within each clonal lineage were isolate-specific, suggests that subpopulations co-evolve from a common ancestor during chronic infection. The presence of diverse subpopulations may favour survival of the bacterial community during high selective pressures. An understanding of how *Achromobacter* sp. establish and maintain chronic infection may contribute to future therapeutic strategies.

## Supporting Information

S1 Fig PFGE results. Pulsed field gel electrophoresis of *XbaI* digested genomic DNA from the 15 *Achromobacter* sp. isolates included in the study. Lanes 1–3: patient CF1, lanes 4–6: patient CF2, lanes 7–9: patient CF3, lanes 10–12: patient CF4, lanes 13–15: patient CF5. Lanes M: molecular marker.
(TIF)

S1 Table Complete list of mutated genes. (XLSX)
