# Supplementary figures and images for: Genetic Adaptation of Achromobacter sp. during Persistence in the Lungs of Cystic Fibrosis Patients
Source: PLoS One. 2015 Aug 27;10(8):e0136790. doi: 10.1371/journal.pone.0136790 (PMC4552427; doi:10.1371/journal.pone.0136790)

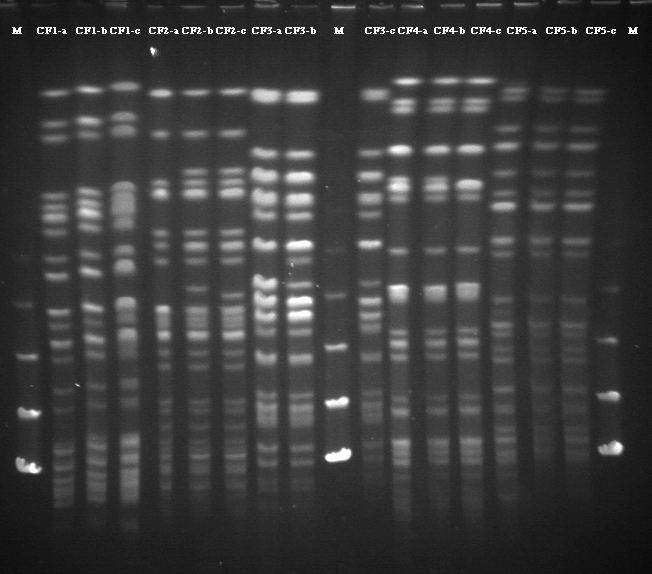

Supplement: S1 Fig — Pulsed field gel electrophoresis of XbaI digested genomic DNA from the 15 Achromobacter sp. isolates included in the study. Lanes 1–3: patient CF1, lanes 4–6: patient CF2, lanes 7–9: patient CF3, lanes 10–12: patient CF4, lanes 13–15: patient CF5. Lanes M: molecular marker. (TIF) [file pone.0136790.s001.tif]
